# NPAS2 promotes aerobic glycolysis and tumor growth in prostate cancer through HIF-1A signaling

**DOI:** 10.1186/s12885-023-10685-w

**Published:** 2023-03-28

**Authors:** Shuaijun Ma, Yafan Chen, Penghe Quan, Jingliang Zhang, Shichao Han, Guohui Wang, Ruochen Qi, Xiaoyan Zhang, Fuli Wang, Jianlin Yuan, Xiaojian Yang, Weijing Jia, Weijun Qin

**Affiliations:** 1 Department of Urology, The First Affiliated Hospital of Air Force Medical University, 127 Changle West Road, 710032 Xi ’an, Shaanxi China; 2 Department of Human Movement Science, Xi’an Physical Education University, Xi’an, China; 3 Department of Hematology, The First Affiliated Hospital of Air Force Medical University, 127 Changle West Road, 710032 Xi ’an, Shaanxi China

**Keywords:** Prostate cancer (PCa), NPAS2, HIF-1A, Glycolysis, Oxidative phosphorylation

## Abstract

**Background:**

Prostate cancer (PCa), one of the common malignant tumors, is the second leading cause of cancer-related deaths in men. The circadian rhythm plays a critical role in disease. Circadian disturbances are often found in patients with tumors and enable to promote tumor development and accelerate its progression. Accumulating evidence suggests that the core clock gene NPAS2 (neuronal PAS domain-containing protein 2) has been implicated in tumors initiation and progression. However, there are few studies on the association between NPAS2 and prostate cancer. The purpose of this paper is to investigate the impact of NPAS2 on cell growth and glucose metabolism in prostate cancer.

**Methods:**

Quantitative real-time PCR (qRT-PCR), immunohistochemical (IHC) staining, western blot, GEO (Gene Expression Omnibus) and CCLE (Cancer Cell Line Encyclopedia) databases were used to analyze the expression of NPAS2 in human PCa tissues and various PCa cell lines. Cell proliferation was assessed using MTS, clonogenic assays, apoptotic analyses, and subcutaneous tumor formation experiments in nude mice. Glucose uptake, lactate production, cellular oxygen consumption rate and medium pH were measured to examine the effect of NPAS2 on glucose metabolism. The relation of NPAS2 and glycolytic genes was analyzed based on TCGA (The Cancer Genome Atlas) database.

**Results:**

Our data showed that NPAS2 expression in prostate cancer patient tissue was elevated compared with that in normal prostate tissue. NPAS2 knockdown inhibited cell proliferation and promoted cell apoptosis in vitro and suppressed tumor growth in a nude mouse model in vivo. NPAS2 knockdown led to glucose uptake and lactate production diminished, oxygen consumption rate and pH elevated. NPAS2 increased HIF-1A (hypoxia-inducible factor-1A) expression, leading to enhanced glycolytic metabolism. There was a positive correlation with the expression of NPAS2 and glycolytic genes, these genes were upregulated with overexpression of NPAS2 while knockdown of NPAS2 led to a lower level.

**Conclusion:**

NPAS2 is upregulated in prostate cancer and promotes cell survival by promoting glycolysis and inhibiting oxidative phosphorylation in PCa cells.

**Supplementary Information:**

The online version contains supplementary material available at 10.1186/s12885-023-10685-w.

## Introduction

Prostate cancer is the most prevalent male malignancy in western countries with high mortality and incidence in males worldwide [1]. This cancer is characterized by extensive molecular heterogeneity and varied clinical outcomes [2], indicating a critical need for increased genetic screening to identify disease-causing variants, which has been already used in the USA and Europe. The following genes were listed with the relative actionability level: BRCA1/2, MSI-H, PTEN, ATM, PALB2, PI3KCA, and AKT. Currently, BRCA1/2 and MSI-H gene mutations represent the alterations with strongest therapeutic actionability and predictivity of therapeutic success [3]. These findings have important implications for prostate cancer diagnosis and screening. The main clinical treatment for prostate cancer includes surgery, hormone therapy, radiotherapy and chemotherapy [4]. Beyond this, large-scale sequencing efforts have allowed a better understanding of the genomic landscape of prostate cancer. Germline or somatic aberrations in the DNA damage repair genes are found in 19% of primary prostate cancer and almost 23% of metastatic castration-resistant prostate cancer and compromise genomic integrity. As such, several PARP inhibitors have been investigated in metastatic castration-resistant prostate cancer patients and are therapeutically effective in germline BRCA2 mutants [5].

Compared with countries in Europe and the Americas, the incidence of prostate cancer is at relatively lower level in China [6]. But with the population aging progress and changes in dietary structure and living environment, prostate cancer incidence continues to increase [7]. This imposes a significant economic burden on patients and healthcare systems. Therefore, it is necessary to find novel markers of prostate cancer to use in diagnosis and treatment. Studies have been reported that androgen receptor variants, bone metabolism, neuroendocrine and metabolite biomarkers were potential serum biomarkers of prostate cancer. Furthermore, in the subset of patients with bone metastases, higher bone sialoprotein levels are related to a shorter time to develop bone metastases in patients with prostate cancer, osteopontin may be of use in assessing treatment response post chemotherapy in patients with castration-resistant prostate cancer [8]. To explore whether similar mechanisms could be detected in prostate cancer patients’ urine is attractive and promising.

Circadian rhythmicity is a fundamental feature of biology, functioning to help organisms pace their physiology and behaviors with the daily light/dark cycle from the Earth’s rotation [9]. The circadian systems are apparently ubiquitous in eukaryotic organisms and have been found in some prokaryotes [10]. In mammals, the circadian system is composed of a central clock in the suprachiasmatic nucleus (SCN) of the hypothalamus. Peripheral clocks locate in extra-SCN regions of the brain as well as in almost all other tissues [11]. Up to now, several core circadian genes have been identified, including *BMAL1, CLOCK, CRY1-2, PER1-3, NPAS2, CSNK1E, NR1D1-2, RORA*, and *TIMELESS* [12]. Circadian rhythms are driven by a positive/negative transcriptional-translational feedback loop generated by core circadian genes [13]. Circadian rhythms modulate many physiological, biological, and behavioral processes in mammals. Recent studies have indicated that abnormal circadian rhythm is related with various types of human diseases, including cancers [14].

*NPAS2*, the largest circadian rhythm gene, heterodimerizes with BMAL1 and regulates the transcription of circadian genes [15]. There is a burgeoning body of evidence suggesting that NPAS2 was involved with carcinoma tumorigenesis and progression. Elevated expression of NPAS2 in hepatocellular carcinoma (HCC) triggers CDC25A transactivation, dephosphorylates Bcl-2, and ultimately inhibits apoptosis [16]. Furthermore, HIF-1BA-mediated reprogramming of glucose metabolism plays a key role in NPAS2-regulated HCC cell growth and metastasis [17]. The rs2305160 in NPAS2 gene was associated to the susceptibility of breast cancer [18]. Epidemiological studies have indicated that circadian rhythm disorder is associated with an enhanced risk of prostate cancer [19]. In our earlier studies, we found increased NPAS2 expression in PCa tissues and cell lines. This regulation promoted glycolysis and proliferation of PCa cells. Thus, in the present study, we sought to reveal the role of NPAS2 in glucose metabolism and cell survival in PCa cells.

## Materials and methods

### Patients tissue samples and public data set collection

Tumor tissues and adjacent non-cancerous normal tissues were obtained from 47 prostate cancer patients who underwent surgery at XiJing Hospital affiliated with Air Force Medical University. The inclusion criteria were: (1) the diagnosis of PCa was confirmed by postoperative histopathological examination. (2) no history of previous radiotherapy or chemotherapy. (3) complete clinical pathology data and follow-up information. (4) no history of malignancy. The patients provided informed consent and this study was approved by the Ethics Committee of Air Force Medical University. The NPAS2 mRNA expression data of PCa tissues and PCa cell lines were downloaded from the TCGA, GEO and CCLE portal. The correlation between glycolytic genes and NPAS2 mRNA expression in PCa tissues was analyzed in the TCGA database.

### Immunohistochemistry (IHC) and hematoxylin-eosin (HE) staining

All the tumor tissues were embedded in paraffin, followed by IHC staining and routine HE staining. The sections were deparaffinized and rehydrated, then placed into the citrate antigen-retrieval solution for antigen retrieval and incubated sequentially with primary and secondary antibodies. The color was developed with DAB and counterstained with hematoxylin. The proportion of positively stained cells was scored according to the following criteria: 0 (< 10% positive cells), 1 (10–25% positive cells), 2 (26–50% positive cells), 3 (51–75% positive cells) and 4 (> 75% positive cells). The intensity of staining was scored as follows: 0 (no staining), 1 (weak staining), 2 (moderate staining) and 3 (strong staining). The final staining score was calculated as the staining intensity score × the proportion score and ranged from 0 to 12.

### Quantitative real-time PCR (qRT-PCR)

The total RNA was extracted from tumor tissues or cells and then reverse transcribed to cDNA for quantitative real-time PCR experiments, the mRNA levels were normalized by β-actin. The primers were synthesized from Sangon Biotech (Shanghai, China). NPAS2 forward primer sequence is 5’-TCTGGATCACAGAGCA

CCTC-3’, and reverse primer sequence is 5’CAGGAGCTCCAGGTCATCA-3’. β-actin forward primer sequence is 5’CCCAGCCATGTACGTTGCTA-3’, and reverse primer sequence is 5’TCACCGGAGTCCATCACGAT-3’. Each sample was replicated three times, and the relative expression level was calculated by the 2^−ΔΔCt^ method.

### Western blot

Cells were collected and lysed with RIPA buffer to extract protein samples. The proteins were run on SDS-PAGE gels and transferred to PVDF membranes. The membranes were blocked with 5% non-fat skim milk, followed by incubation with primary antibodies at 4 °C overnight and incubated by secondary antibody for 1 h at room temperature. Ultimately, the specific bands were detected using ECL chromogenic solution. The intensity of bands was performed using Image J software. Anti-NPAS2 antibody was purchased from NOVUS (USA), other antibodies were commercially purchased from Proteintech (Wuhan, China).

### Cell cultures and transfection

The human prostate cancer cell lines (PC-3, MDA PCa 2, DU145,22RV1 and C4-2) and human prostate epithelial cell line (RWPE-1) were purchased from the American Type Culture Collection ( Manassas, Virginia, USA) and the Cell Bank of the Chinese (Shanghai, China). These cells were cultured in RPMI-1640 medium supplemented with 10% FBS, 1% penicillin/streptomycin and incubated at 37 °C in a 5% CO_2_. All cell lines were recently authenticated by cellular morphology and short tandem repeat profiling. NPAS2 overexpression lentivirus was stably transfected into C4-2 cell lines and an empty vector (EV) served as a negative control. For the NPAS2 knockdown, PC-3 cells were stably transfected with shNPAS2 lentivirus, and the corresponding control group was called shCtrl. The transfection effects were validated by qRT-PCR and western blot.

### MTS assay

Cell viability was determined by MTS (Bestbio, Shanghai) assay. Cells were seeded in triplicate at 5000 cells per well in 100 µL complete culture media in a 96-well microplate and cultured overnight at 37 °C with 5% CO_2_, 10 µl MTS were added to each well, and the cells were incubated for another 1–4 h. Absorbance was measured by a microplate reader at 490 nm. The cell viability was calculated using the formula: cell viability (%) = 100× (OD sample – OD blank)/ (OD control – OD blank).

### Colony formation assay

1,000 cells were plated in 6-well plates and cultured for approximately two weeks. Each experiment had three repeat wells. Finally, the number of clones in each well was then counted.

### 5-Ethynyl-2′-Deoxyuridine (EdU) assays

A total of 3 × 10^4 cells per well were seeded into 96-well plates and treated with 50 µM EdU (Ribobio, Guangzhou) for 2 h. Living cells were fixed in 4% phosphate-buffered paraformaldehyde, neutralized by 2 mg/mL glycine and subjected to permeabilization in 0.5% Triton X-100. Following Apollo staining and DAPI staining, a fluorescent microscope was adopted to observe the EdU positive cells.

### Apoptosis detection

Cells were collected and rinsed with cold PBS, then stained using Annexin V-FITC/PI (Bestbio, Shanghai) and apoptosis was detected by flow cytometry.

### Measurement of glucose uptake, lactate production, pH and oxygen consumption rate

The medium was collected to detect glucose uptake, lactate production and pH. The glucose uptake and lactate production were assessed with glucose uptake assay kit and lactate assay kit (Sigma, USA) following manufacturer’s instructions. The oxygen consumption rate was measured using a Hansatech Oxytherm (Hansatech, King’s Lynn, UK).

### Xenograft tumor model in nude mice

Male nude mice (BALB/C Nude, 3–5 weeks old) were purchased from the Animal Experiment Center of Air Force Medical University. They were housed in a specific pathogen free (SPF) facility and treated according to standard protocols and animal welfare regulations. Nude mice were acclimatized to the laboratory conditions for 2–3 days prior to the beginning of the experiments. This study was approved by the Ethics Committee of Air Force Medical University. The mice were randomly assigned to groups in the experiment (shCtrl: 5 nude mice, shNPAS2: 5 nude mice). About 1 × 10^7 cells (200 µL) were injected subcutaneously into the back of BALB/C nude mice. The growth and diet of nude mice were observed, the volume of subcutaneous tumors was measured every three days. Tumor volume was calculated (volume = (width)2 × length/2). At the end of the experiments, the tumors were excised and weighed, then fixed in 4% paraformaldehyde and paraffin-embedded. ARRIVE guidelines (http://arriveguidelines.org) were followed.

### Statistical analysis

For all studies, data were analyzed using GraphPad Prism 9.0 software (GraphPad, La Jolla, CA, USA) and are presented as the mean ± standard error of the mean (SEM). The paired t-test was used to compare the quantification results of immunohistochemistry. Comparison of more than two samples already have a first approach by ANOVA or equivalent non-parametric method before two-by-two sample comparison. Unpaired t tests were used for comparisons between 2 groups where appropriate. Statistical significance was considered if *P* < 0.05. Each experiment was repeated at least three times.

## Results

### NPAS2 is significantly upregulated in prostate cancer

**Fig. 1:**
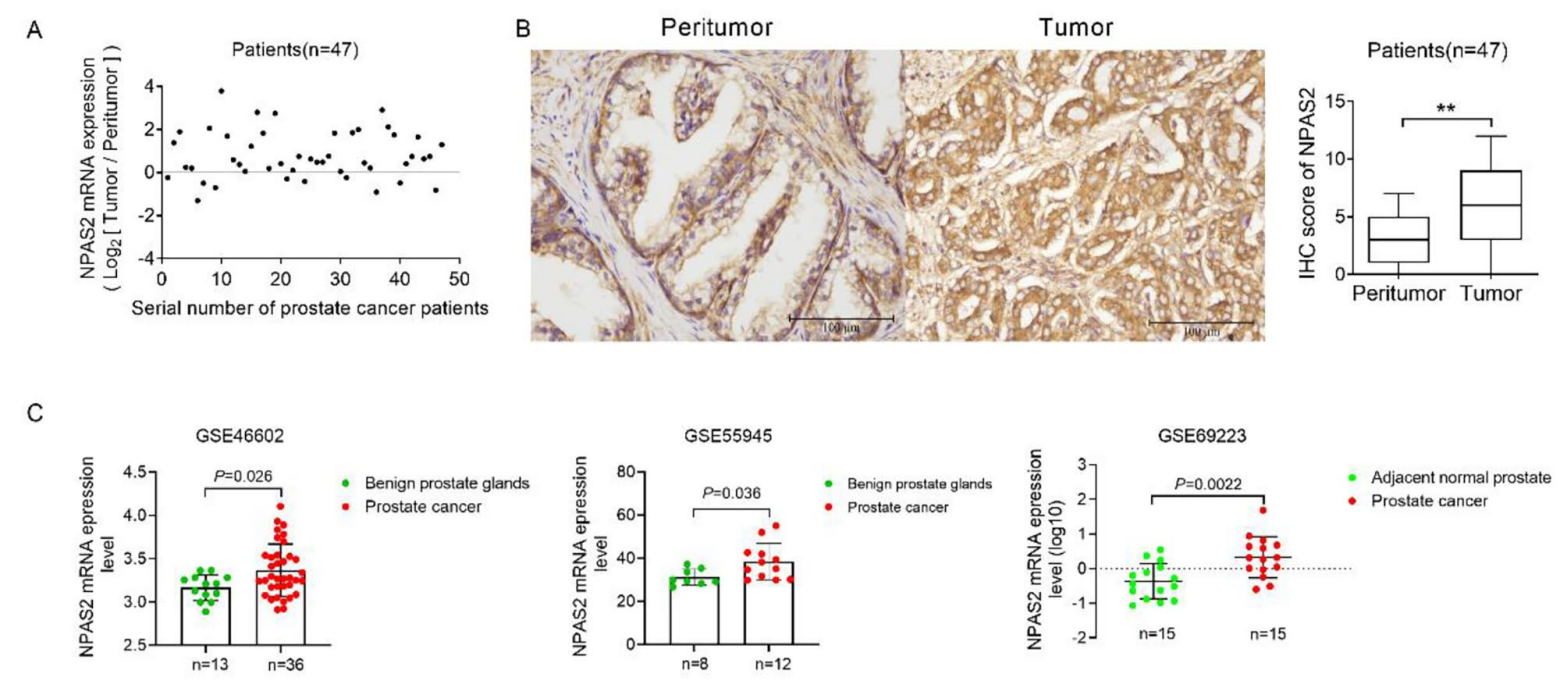
NPAS2 is significantly upregulated in prostate cancer (A) qRT-PCR analysis the expression of NPAS2 mRNA in 47 PCa tissues (B) IHC analysis the expression of NPAS2 in 47 PCa tissues. Scale bar, 100 μm (C) Bioinformatics analysis of NPAS2 mRNA expression level in prostate cancer GSE46602, GSE55945 and GSE69223 databases.*P＜0.05; **P＜0.01

To observe the expression of the core rhythm gene NPAS2 in prostate cancer, we firstly analyzed the expression of NPAS2 in 47 PCa tissues and corresponding paraneoplastic tissues by qRT-PCR and IHC. The results showed that NPAS2 expression in prostate cancer patient tissue was elevated compared with that in normal prostate tissue (Fig. 1A and B). Afterwards, we took advantage of the TCGA and GEO databases and found that prostate tumors express significantly higher levels of NPAS2 mRNA than in adjacent normal tissues and benign prostate samples (Fig. 1C).

### NPAS2 promotes PCa cell survival in vitro

**Fig. 2:**
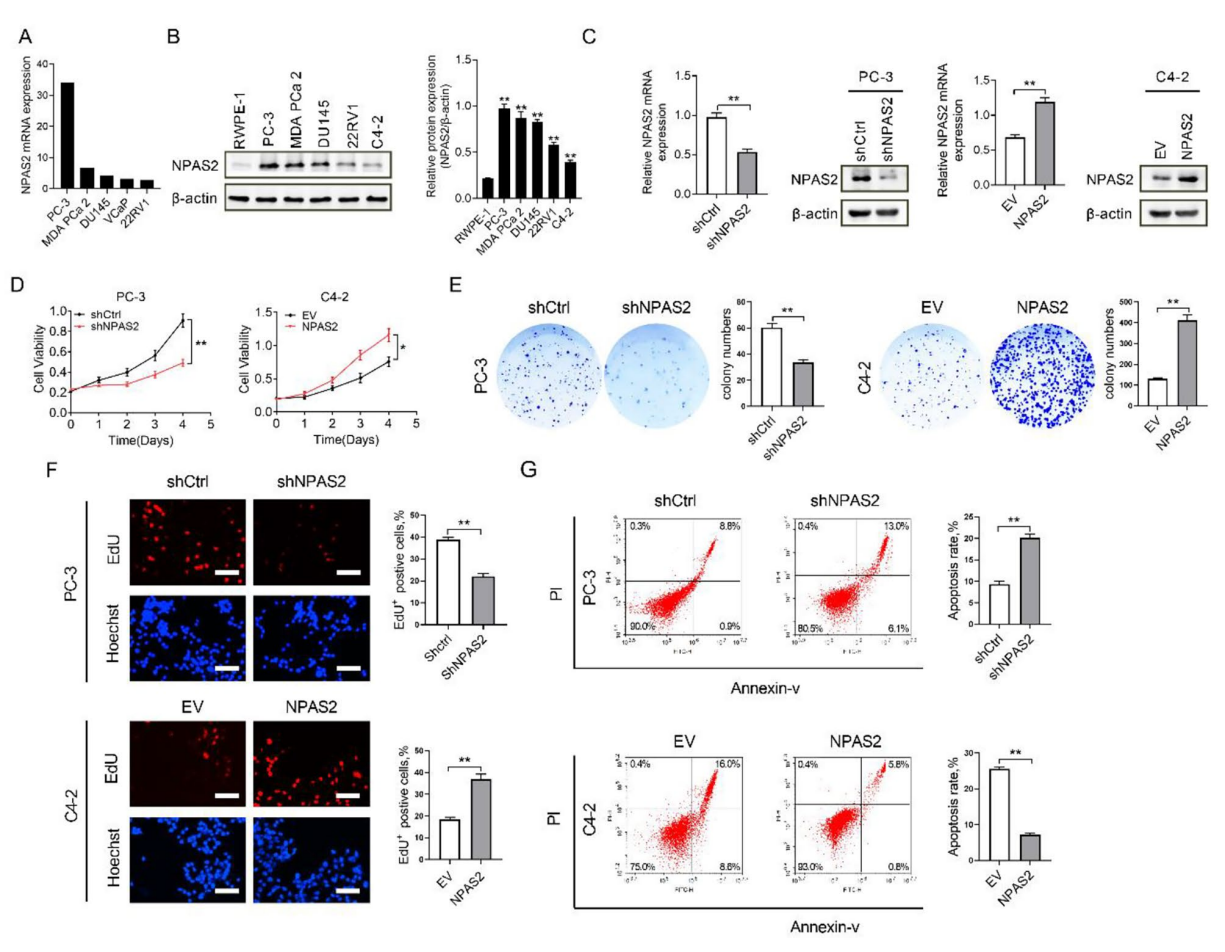
**NPAS2 promotes PCa cell survival in vitro** (A) Expression of NPAS2 in PCa cell lines in the CCLE database (B) Western blot analysis of NPAS2 expression in PCa cell lines and a normal prostate epithelial cell (C) NPAS2 expression levels in the transfected PCa cell lines were detected through western blot and qRT-PCR analysis (D) MTS growth experiments in cell transfection models (E) Colony formation experiments in cell transfection models (F) EdU proliferation assay in cell transfection models. Scale bar: 50 μm (G) Apoptosis detection in cell transfection models. *P＜0.05; **P＜0.01. Data were shown as the mean ± S.E.M. from three independent experiments

After found that NPAS2 was upregulated in PCa tissues, we further explored the role of NPAS2 in PCa cells. NPAS2 mRNA was differentially expressed in PCa cell lines based on the CCLE database (Fig. 2A). Western blot and qRT-PCR analysis showed that the expression of NPAS2 in five PCa cell lines (PC-3, MDA PCa 2, DU145, 22RV1, C4-2) was significantly higher than in a normal prostate epithelial cell line (RWPE-1) (Fig. 2B). Then we selected C4-2 cells with relatively low NPAS2 expression for overexpression of NPAS2 and PC-3 cells with relatively high NPAS2 expression to knockdown of NPAS2 expression. We transfected C4-2 cells with NPAS2 overexpressing lentivirus and PC-3 cells with the shNPAS2 lentivirus, stable cell lines were selected and confirmed by western blot and qRT-PCR (Fig. 2C). MTS cell viability and colony formation assays indicated that knockdown of NPAS2 significantly reduced cell viability and colony formation in PC-3 cells. However, the opposite trend was observed in C4-2 cells with NPAS2 overexpression (Fig. 2D and E). Subsequently, EdU test detected the proliferation of cells in each group, red colors indicated represent proliferating cells (EdU-positive cells). Our EdU assay results suggested that the cell proliferation activity in the shNPAS2 groups was marbly inhibited compared with the shCtrl group and overexpression of NPAS2 obviously enhanced proliferation of C4-2 cells (Fig. 2F). In addition to increasing cell proliferation, we also wanted to know whether the expression of NPAS2 could affect cellular apoptosis. Results demonstrated that knocking down NPAS2 increased early apoptotic cells and late apoptotic cells, meanwhile NPAS2 overexpression led to decreased apoptosis (Fig. 2G). These findings collectively indicated that NPAS2 could facilitate cell proliferation and inhibit cell apoptosis.

### NPAS2 knockdown inhibits PCa tumor growth in vivo

**Fig. 3:**
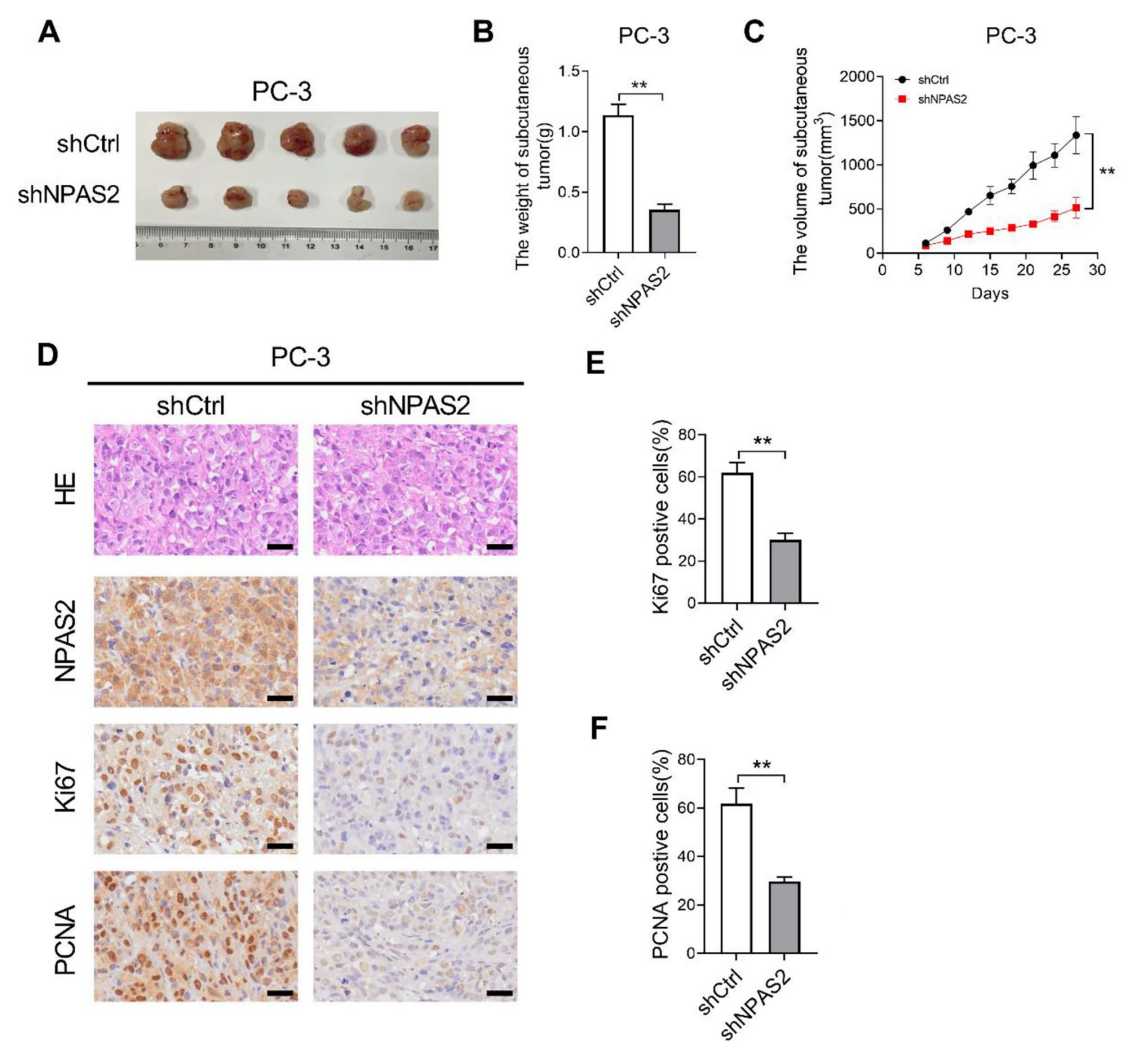
**NPAS2 knockdown inhibits PCa tumor growth in vivo** (A) Subcutaneous xenograft model for PC-3 cells treated as indicated and dissected tumors from sacrificed mice were shown (B) Weight of tumors in nude mice (C) Tumor volume changes curve of nude mice (D) HE staining, immunohistochemical staining of NPAS2, Ki67 and PCNA in nude mice tumors. Scale bar: 50 μm (E) Comparison of Ki-67-positive cells in tumor tissues of nude mice xenograft model with different treatment as indicated (F) Comparison of PCNA-positive cells in tumor tissues of nude mice xenograft model with different treatment as indicated. *P＜0.05; **P＜0.01

With the aim of investigating the effects of NPAS2 on prostate tumor growth in vivo, we subjected the PC-3 cells with a stable NPAS2 knockdown to nude mice, these tumor cells formed a relatively obvious tumor cell mass (Fig. 3A). Compared with the shCtrl group, the NPAS2 knockdown nude mice showed a significant reduction in tumor volumes and tumor weights (Fig. 3B and C). The expressions of NPAS2, Ki67 and PCNA in tumor tissues of nude mice were tested by immunohistochemistry. Of note, when compared with controls, those xenografts developed from PC-3 cells with NPAS2 stable knockdown exhibited a considerable decrease of positive Ki-67 and PCNA staining (Fig. 3D-F). These results revealed that knockdown of NPAS2 could suppress the growth of PCa tumors in vivo.

### NPAS2 promotes glycolysis and inhibits oxidative phosphorylation in PCa cells

**Fig. 4:**
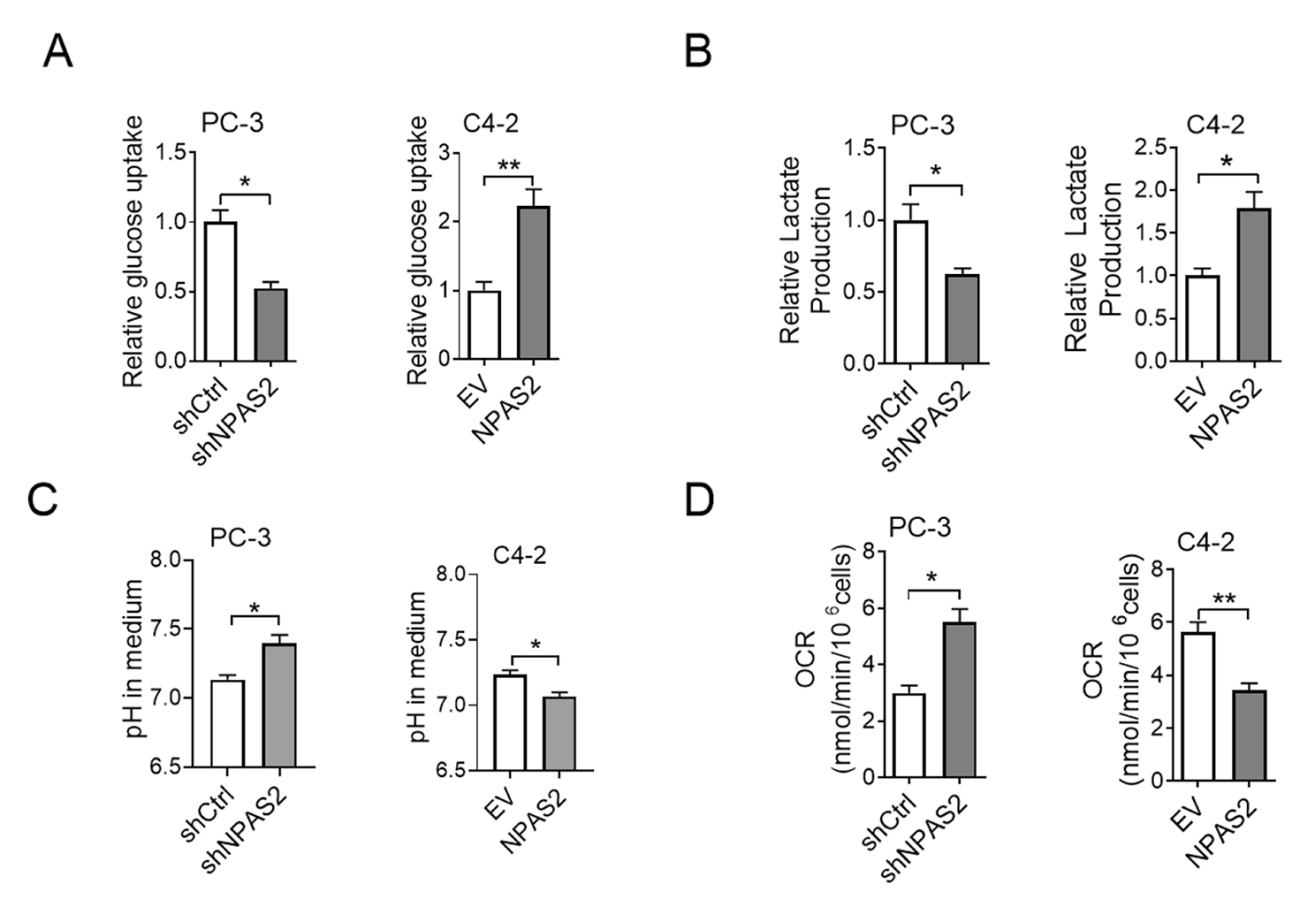
**NPAS2 promotes glycolysis and inhibits oxidative phosphorylation in PCa cells** (A) Glucose uptake in cell transfection models (B) Lactate production in cell transfection models (C) Medium pH of cell transfection models (D) Oxygen consumption level of cell transfection models. *P＜0.05; **P＜0.01. Data were shown as the mean ± S.E.M. from three independent experiments

Glucose serves as a main nutrient to fuel cellular metabolic activities. Cell proliferation could be enhanced by metabolic changes. Based on the stable cell lines, some glucose metabolism indicators of PCa cells in PC-3 and C4-2 were examined. We observed that NPAS2 knockdown in PC-3 cells led to glucose uptake and lactate production diminished, NPAS2 overexpression significantly advancing glucose uptake and lactate production (Fig. 4A and B). Subsequently, we detected the oxygen consumption rate and pH, and the results demonstrated that the knockdown of NPAS2 elevated oxygen consumption rate and pH in PC-3 cells, while NPAS2 overexpression reduced oxygen consumption rate and pH in C4-2 cells (Fig. 4C and D). These phenotypes demonstrated the NPAS2 could enhance glycolysis in PCa cells. Taken together, NPAS2 regulated glucose metabolism in PCa cells by promoting glucose uptake and glycolysis and inhibiting oxidative phosphorylation.

**Fig. 5:**
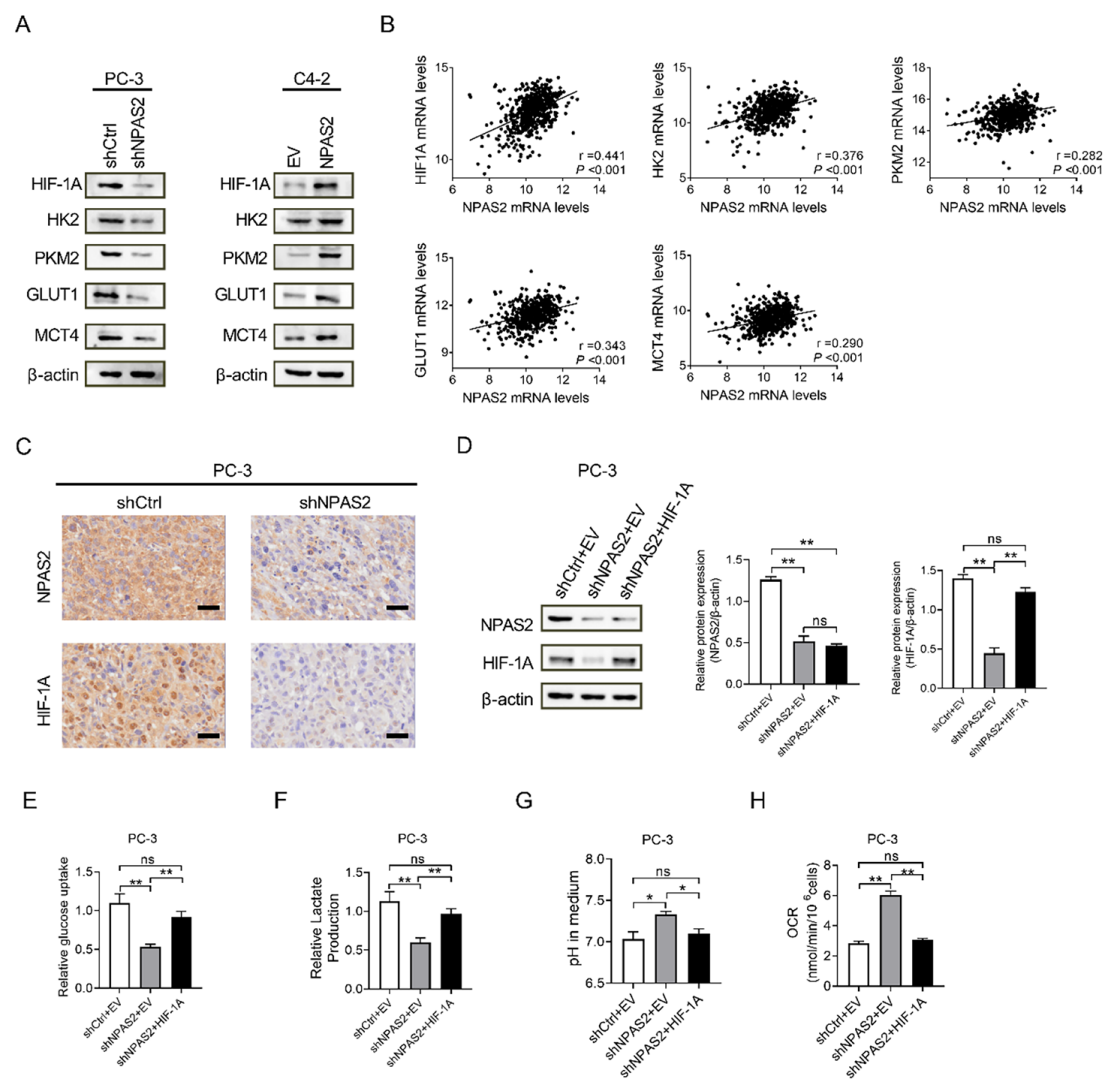
**NPAS2 enhances glycolysis by increasing the expression of key molecules in the glycolytic pathway** (A) Western blot analysis of the expression levels of NPAS2 and key glycolysis genes in PCa cells (B) Correlation between NPAS2 expression and key molecules of glucose metabolism in prostate cancer tissues (TCGA database) (C) Immunohistochemical staining of NPAS2 and HIF-1A in nude mice tumors (D) The transfection efficiency was examined with western blot (ANOVA, *P*<0.0001) (E) Glucose uptake in cell transfection models (ANOVA, *P* = 0.007) (F) Lactate production in cell transfection models (ANOVA, *P* = 0.012) (G) Medium pH of cell transfection models (ANOVA, *P* = 0.036) (H) Oxygen consumption level of cell transfection models (ANOVA, *P*<0.0001). *P＜0.05; **P＜0.01. Data were shown as the mean ± S.E.M. from three independent experiments

### NPAS2 enhances glycolysis by increasing the expression of key molecules in the glycolytic pathway

Based on the above cell phenotype results, we speculated that NPAS2 could be related to glycolysis and oxidative phosphorylation in PCa cells. A series of investigations indicated that transcription factors HIF-1A played critical roles in the regulation of both glycolysis and oxidative phosphorylation [20, 21]. Our results shown that in PCa cells HIF-1A was upregulated with overexpression of NPAS2 while knockdown of NPAS2 led to a lower level (Fig. 5A). Following that, we examined the correlation between HIF-1A and NPAS2 mRNA expression in prostate cancer tissues in the TCGA database. The database searched a positive correlation of the mRNA expression of HIF-1A with NPAS2 mRNA levels (Fig. 5B). Subsequently, judging from the immunohistochemistry results, the HIF-1A expression was profoundly reduced by knockdown of NPAS2 in nude mice tumors (Fig. 5C). The results above indicated that NPAS2 might promote glycolysis by increasing HIF-1A expression. To test this idea, we transfected HIF-1A overexpression lentivirus to PC-3 shCtrl and shNPAS2 cells, western blot analysis was used to verify the transfection effects (Fig. 5D). Knockdown of NPAS2 decreased glucose uptake and lactate production in PCa cells, while overexpression of HIF-1A generally restored the inhibitory effect (Fig. 5E and F). Increasing of oxygen consumption rate and pH were shown after NPAS2 knockdown in PC-3 cells, while overexpression of HIF-1A generally suppressed the promoting effect (Fig. 5G and H).

Furthermore, we explored whether NPAS2 was involved in HIF-1A-mediated upregulation of the glycolytic genes of HK2, PKM2, GLUT1 and MCT4. As shown in Fig. 5A, forced expression of NPAS2 significantly increased the expression of HK2, PKM2, GLUT1 and MCT4 which were downregulated by NPAS2 knockdown. Bioinformatics analysis based on TCGA public mRNA expression datasets also showed a positive correlation between NPAS2 and the above-mentioned genes (Fig. 5B). The results above indicated that the mechanisms by which NPAS2 affects PCa glycolytic regulation are achieved through HIF-1A.

## Discussion

Most tumor cells exhibit a high rate of glycolysis even under conditions of ample oxygen supply [22–24]. Despite accumulating evidence has been presented that there is a close relation between circadian rhythm and tumorigenesis [25]. The role of NPAS2 in PCa cells glucose metabolic reprogramming is poorly understood. Metabolic reprogramming is one of the main characteristics of tumors, while glycolysis being the foremost manifestation of metabolic reprogramming, playing a pivotal role in tumorigenesis and progress. In this study, we discover that NPAS2 expression is elevated in PCa tissues and cells. The high expression of NPAS2 promotes the release of HIF-1A and key glycolytic genes (HK2, PKM2, GLUT1 and MCT4), enhancing glycolytic metabolite levels, thus inducing the progression of prostate cancer cells.

The circadian system has strong influences on glucose metabolism, circadian disruption increases the risk of metabolic syndrome and type 2 diabetes [26]. Circadian rhythms also govern an extensive variety of behavioral, physiological and metabolic functions in virtually all life forms in mammalian [27]. However, whether circadian genes regulate the metabolism reprogramming remains largely unknown. In the current study, we find that NAPS2 is involved in the aerobic glycolysis in PCa cells. Aerobic glycolysis can increase the lactate content of cancer cells and reduce the pH of the tumor microenvironment. The elevated aerobic glycolysis rate can be caused by multiple mechanisms in cancer cells, typically accompanied by key glycolytic genes increased. Our findings indicate that NPAS2 promotes glycolysis of PCa cells by up-regulating HIF-1A, HK2, PKM2, GLUT1 and MCT4.

A major regulator of glycolytic activity in tumors is the transcription factor HIF-1A. HIF-1A plays a critical role in aerobic glycolysis through activating its downstream factor [28–30]. Activation of HIF-1A is one of the most common features in human cancers [31]. HIF-1 can produce pleiotropic effects on cancer cells and stromal cells, such as promote vascularization. Cells react to hypoxia via the regulation of HIF. Hypoxia inducible factors (HIF) are heterodimeric proteins belonging to the basic helix-loop-helix transcription factor families, composed of an alpha and a beta subunit (HIF-1A and HIF-b). Under normoxic conditions, HIF-1A are hydroxylated by prolyl hydroxylases, which causes them to be ubiquitinated by VHL, thereby targeting HIF-1A for proteasomal degradation. Under hypoxic conditions, HIF-1A subunits are stabilized due to a lack of hydroxylation. HIF-1A can then dichotomize with HIF-b and prompt the transcription of hypoxia-survival genes. Among the transcripts managed by HIF-1 is VEGF, which permits tissues to adjust to a hypoxic environment by enhancing angiogenesis [32].

Previous studies have identified many polymorphisms affecting multiple clock genes (BMAL1, CLOCK and NPAS2) that are linked to increased risks in prostate, breast, ovarian and pancreatic cancers [33, 34]. But the specific role of NPAS2 during PCa is less well studied. Our current study suggests that NPAS2 is overexpressed in PCa. Tumor-promoting function of NPAS2 is dependent on HIF-1A activation and the engagement of glycolysis-related genes, indicating that NPAS2 may serve as an important therapeutic target to normalize glucose metabolic aberrations responsible for PCa progression.

In summary, our findings indicate that a key regulatory role of NPAS2 in glucose metabolism of PCa cells, which presents novel insights to help understand the mechanisms of circadian dysfunction in PCa progression and find new target for treatment of this malignancy.

## Conclusion

NPAS2 is upregulated in prostate cancer and promotes cell survival by promoting glycolysis and inhibiting oxidative phosphorylation through HIF-1A signaling in PCa cells.

## Electronic supplementary material

Below is the link to the electronic supplementary material.

Supplementary Material 1

## Data Availability

The data applied in the bioinformatics analysis were obtained from TCGA CCLE and GEO mRNA open-access database. The datasets used and/or analyzed during the current study are available from the corresponding author on reasonable request.

## Abbreviations

PCa: Prostate cancer
NPAS2: Neuronal PAS domain-containing protein 2
qRT-PCR: Quantitative real-time PCR
IHC: Immunohistochemistry
CCLE: Cancer Cell Line Encyclopedia
TCGA: The Cancer Genome Atlas
GEO: Gene Expression Omnibus
HIF-1A: Hypoxia-inducible factor-1A
