# Supplementary material for: NPAS2 promotes aerobic glycolysis and tumor growth in prostate cancer through HIF-1A signaling
Source: BMC Cancer. 2023 Mar 28;23:280. doi: 10.1186/s12885-023-10685-w (PMC10045944; doi:10.1186/s12885-023-10685-w)
Supplement: Supplementary file 1 — Supplementary Material 1 [file 12885_2023_10685_MOESM1_ESM.pptx]

## Slide 1
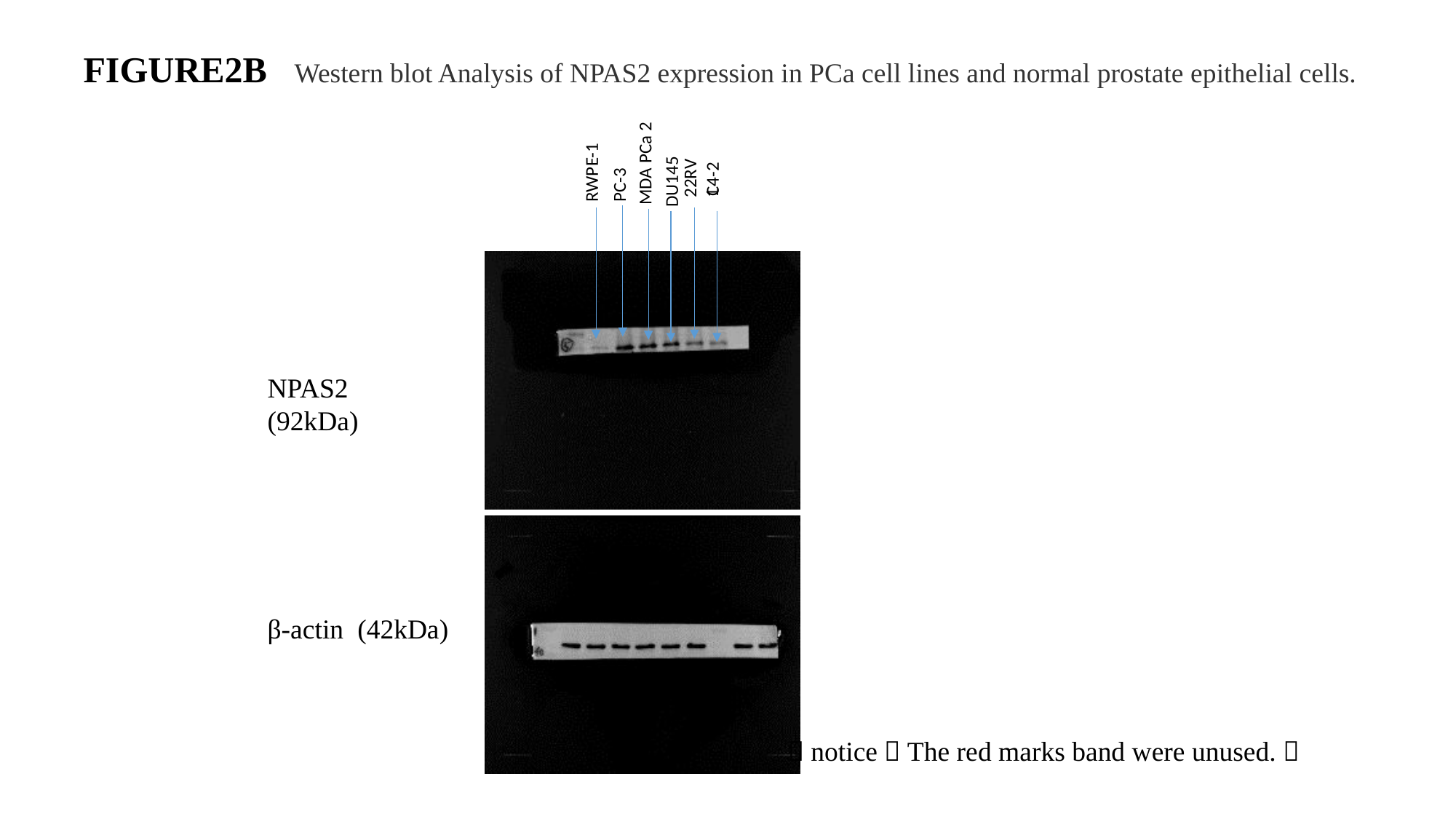

FIGURE2B Western blot Analysis of NPAS2 expression in PCa cell lines and normal prostate epithelial cells.
MDA PCa 2
RWPE-1
DU145
C4-2
22RV1
PC-3
NPAS2 (92kDa)
β-actin (42kDa)
（notice：The red marks band were unused.）

## Slide 2
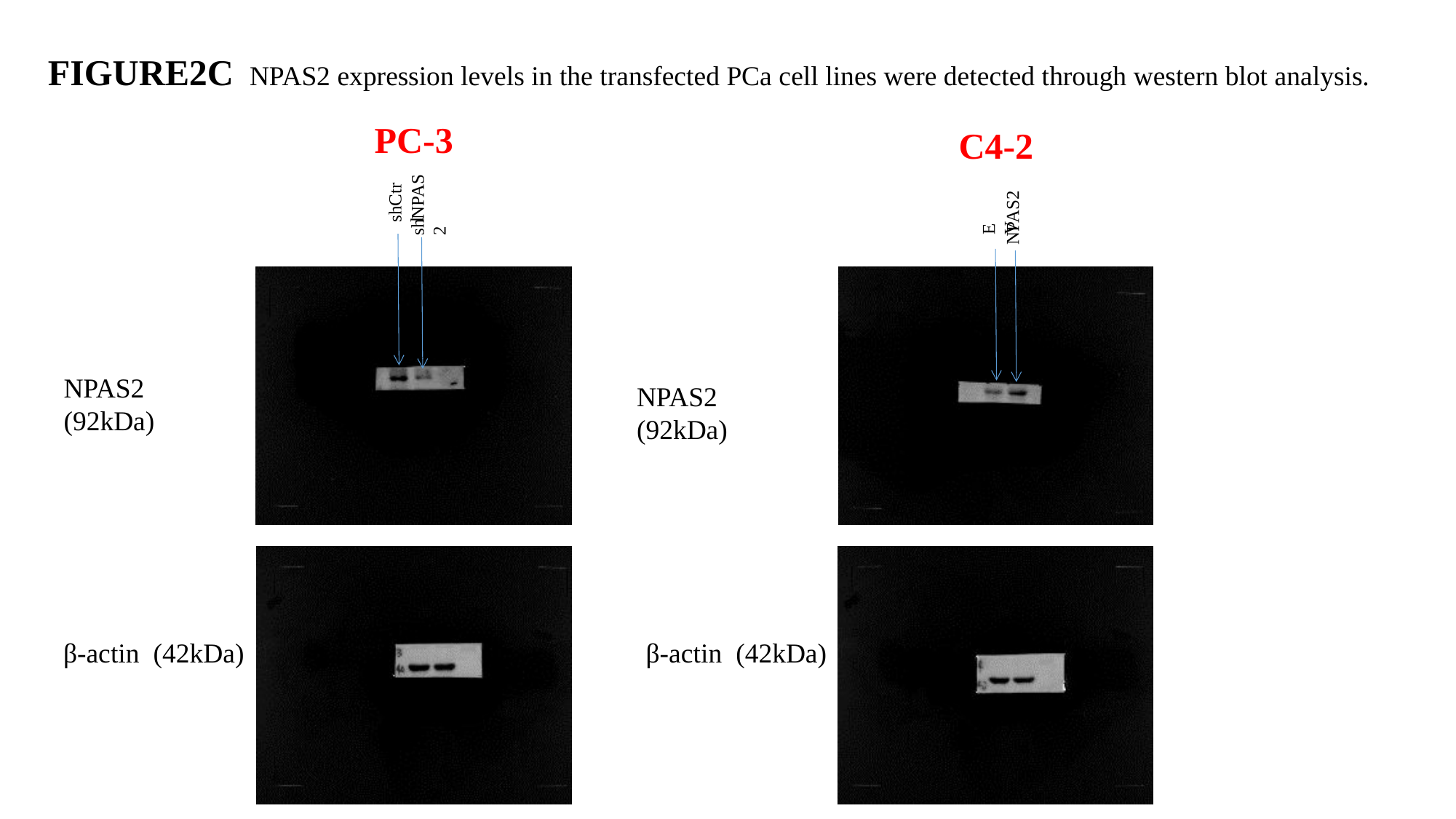

FIGURE2C NPAS2 expression levels in the transfected PCa cell lines were detected through western blot analysis.
PC-3
C4-2
shNPAS2
shCtrl
NPAS2
EV
NPAS2 (92kDa)
NPAS2 (92kDa)
β-actin (42kDa)
β-actin (42kDa)

## Slide 3
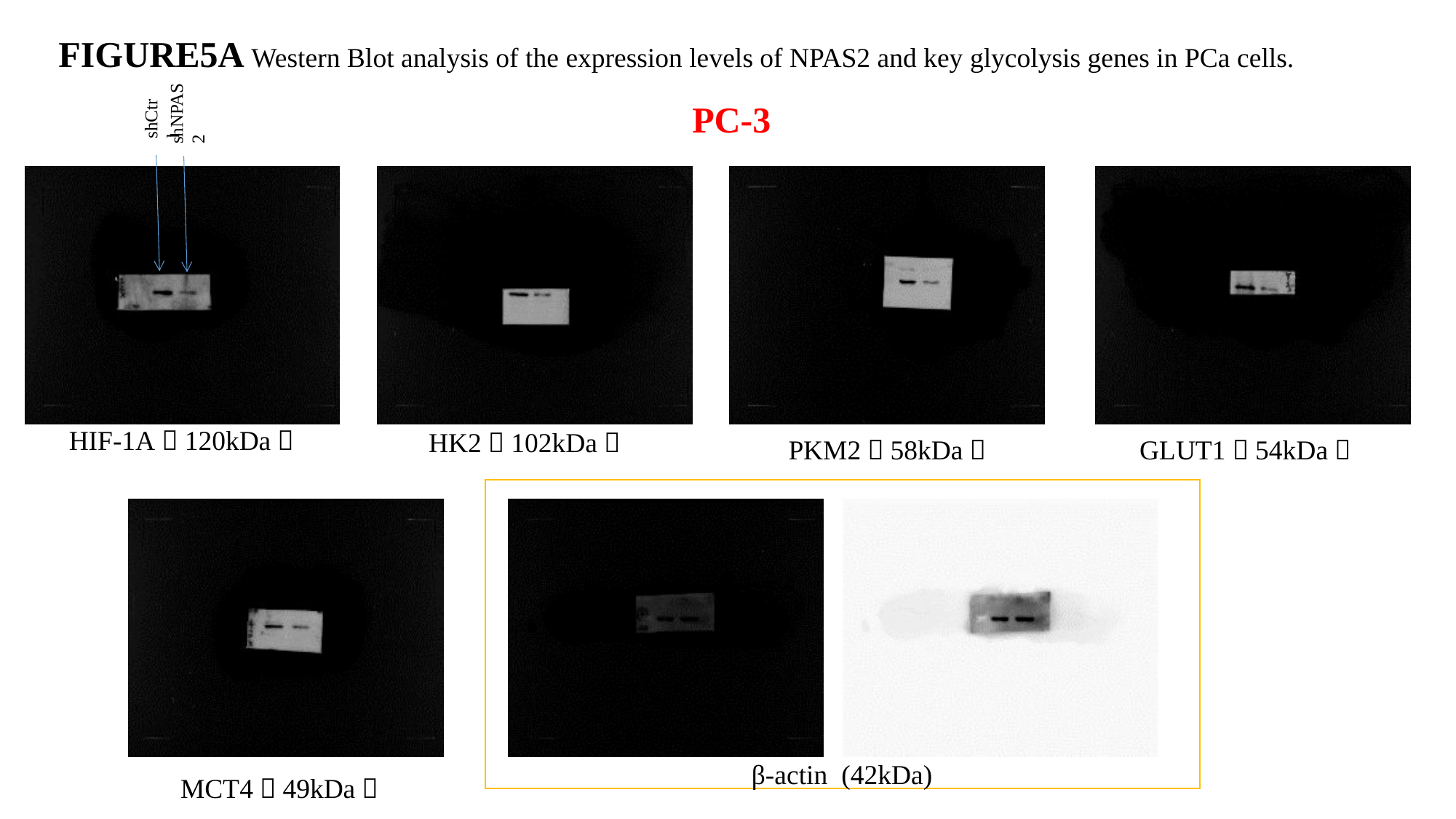

FIGURE5A Western Blot analysis of the expression levels of NPAS2 and key glycolysis genes in PCa cells.
shNPAS2
shCtrl
PC-3
HIF-1A（120kDa）
HK2（102kDa）
PKM2（58kDa）
GLUT1（54kDa）
β-actin (42kDa)
MCT4（49kDa）

## Slide 4
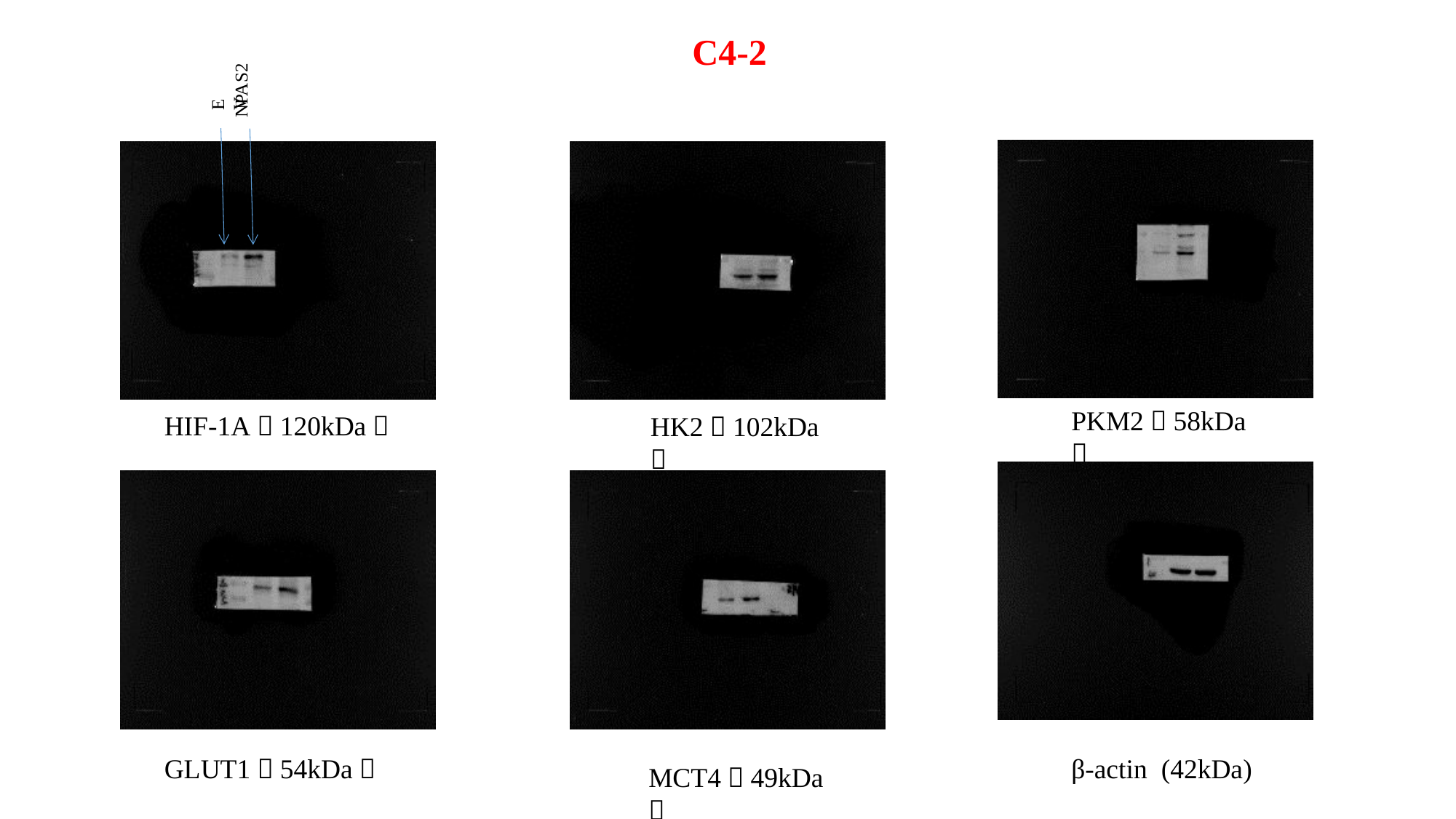

C4-2
NPAS2
EV
PKM2（58kDa）
HIF-1A（120kDa）
HK2（102kDa）
GLUT1（54kDa）
β-actin (42kDa)
MCT4（49kDa）

## Slide 5
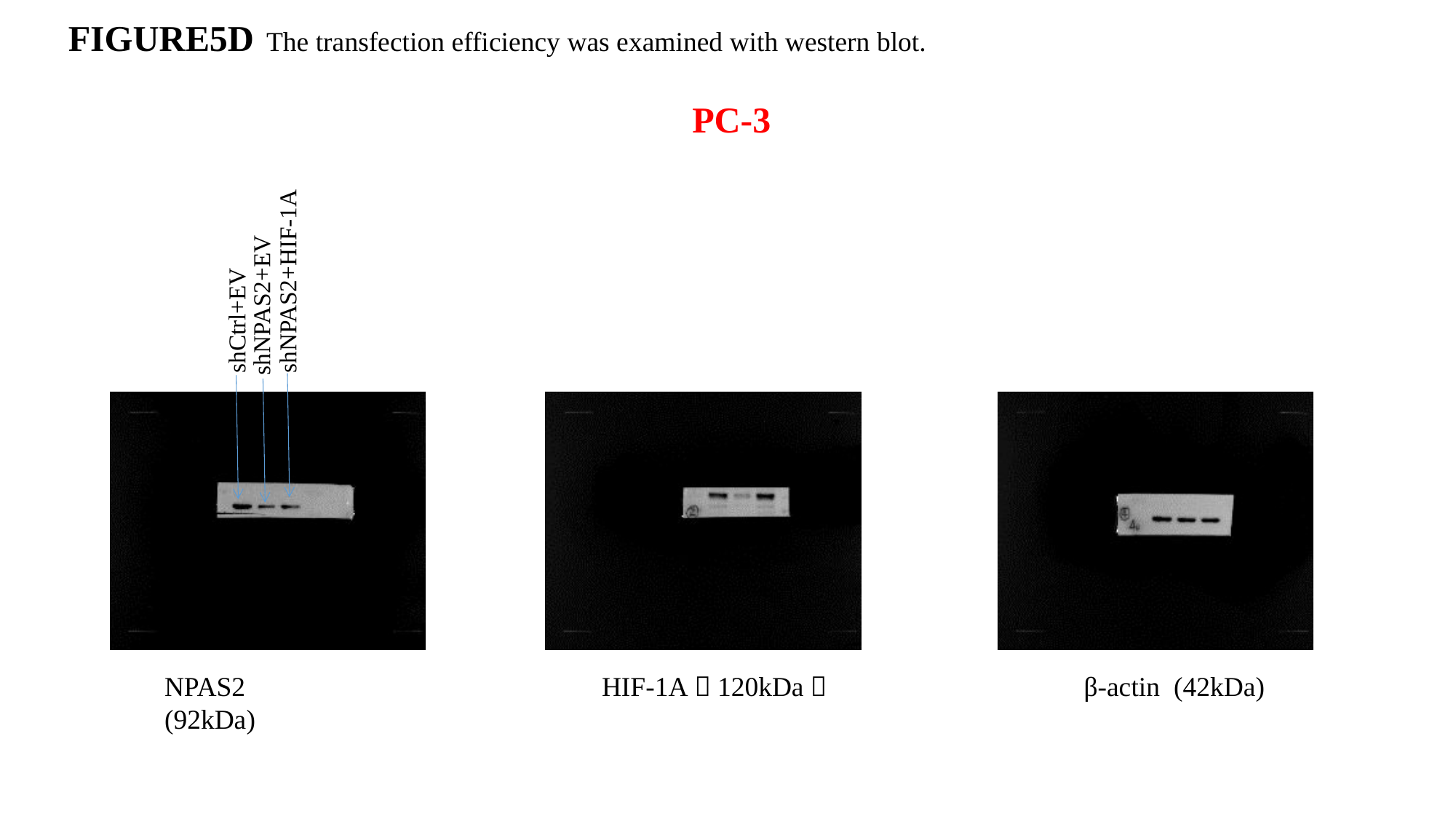

FIGURE5D The transfection efficiency was examined with western blot.
PC-3
shNPAS2+HIF-1A
shCtrl+EV
shNPAS2+EV
HIF-1A（120kDa）
β-actin (42kDa)
NPAS2 (92kDa)
